# Socio-economic class, rurality and risk of cutaneous melanoma by site and gender in Sweden

**DOI:** 10.1186/1471-2458-8-33

**Published:** 2008-01-25

**Authors:** Beatriz Pérez-Gómez, Nuria Aragonés, Per Gustavsson, Virginia Lope, Gonzalo López-Abente, Marina Pollán

**Affiliations:** 1 Cancer and Environmental Epidemiology Unit, National Centre for Epidemiology, Carlos III Institute of Health, Madrid, Spain; 2 Consortium for Biomedical Research in Epidemiology & Public Health (CIBER en Epidemiología y Salud Pública – CIBERESP), Carlos III Institute of Health, Madrid, Spain; 3 Division of Occupational and Environmental Medicine, Department of Public Health Sciences, Karolinska Institutet, Stockholm, Sweden

## Abstract

**Background:**

Cutaneous melanoma (CM) is a cancer usually associated with high socio-economic level in the literature. Few studies have, however, assessed this relationship by gender and site or the association between CM and rurality.

**Methods:**

A major-sized historical occupational Swedish cohort comprising 2,992,166 workers was used to estimate relative risk of cutaneous melanoma, broken down by gender and anatomical site, for occupational sectors (as a proxy of socio-economic class) and rurality. To this end, Poisson models were fitted for each site in men and women, including occupational sector and town size, with adjustment for age, period of diagnosis and geographical area as possible confounding factors.

**Results:**

White collar workers presented a marked increased of risk in men in all melanoma cases, as well as in trunk, upper and lower limbs. This pattern was less clear for women, in which some heterogeneity appeared, as low risks in lower socioeconomic sectors in trunk, or risk excesses in white collar workers in lower limbs did not achieve statistical significance. Males also showed significant differences in risk by rural/urban distribution, but in women this association was limited to CM of lower limb. Risk of CM of head/neck did not vary by occupational sector or town size, thus depicting a specific epidemiological profile, which proved common to both sexes.

**Conclusion:**

While differences in risk between men and women could suggest greater homogeneity in UV-exposure behaviour among women, the uniform risk pattern in head and neck melanoma, present in both sexes, might support the coexistence of different aetiological pathways, related to anatomical site.

## Background

Cutaneous melanoma (CM) is one of the neoplasms more usually associated with higher social class [1-5]. As ultraviolet radiation (UV) is the main aetiological agent for CM, this relationship has been attributed to differences in UV-exposure-related behaviour. The sun is considered responsible for almost 65% of cases [6], mainly through intermittent exposure [7] during summer holidays, something that tends to be more usual among persons having a higher socio-economic class [2] and residing in larger towns [8]. Sun-bed use [9] has also been related to increased CM risk; and other lifestyle-related exposures in which social class might differ, such as occupation [10,11], tobacco or alcohol use [12], diet [13], or contraceptive use [14], have been studied as possible modifiers of melanoma risk, albeit with inconclusive results.

Nevertheless, most studies on socio-economic level and melanoma have mainly focused on men, and anatomical site has not usually been addressed [4,15]. Several authors have suggested the coexistence of different site-related biological pathways [16] that could possibly lead to melanoma. Hence, as reported [17-20], risk factors might conceivably differ by site, which would justify the interest in studying risk factors for each anatomical location separately.

Using a major-sized occupational Swedish historical cohort [21], comprising almost 3 million persons followed up from 1971 to 1989, we studied the relationship between occupational sector (a surrogate indicator of socio-economic class), rurality and melanoma incidence by site and sex, duly adjusted for age, period of diagnosis and latitude. The homogeneity of the population and the relatively low immigration rate across the study period served to reduce any possible confounding due to racial patterns.

## Methods

This historical cohort was made up of 1,890,497 Swedish men and 1,101,669 women employed at the time of the 1970 census, present in the 1960 census, alive and aged 25–64 years on 1 January 1971, and followed up until the end of 1989. Whereas two thirds of the female cohort members were full-time workers – 23% working 20–34 hours and 11% working under 20 hours per week – 97% of males were full-time workers – 2% working 20–34 hours and 0.9% working under 20 hours per week.

Information was drawn from the following two linked data sets [22]: 1) the Swedish Cancer Environment Register, with information on incident cases, used to compute specific rate numerators; and 2) a background population register comprising all individuals in the 1970 census, with information on occupation and residence in 1970, and, where applicable, date of death, used to calculate specific rate denominators. Melanoma was coded under rubric 190 of the International Classification of Diseases (7^th ^revision), in which the fourth digit specifies body site. *In situ *melanomas were included. All head and neck melanomas were analysed jointly. Cutaneous melanomas with multiple or unspecified sites respectively represented 0.7% and 10.6% of all registered melanomas in men, and 0.4% and 9.8% in women. These cases were not included in the site-specific analysis.

Occupational sectors were used as surrogate indicators of social class. In the Nordic Classification of Occupations, each occupation is represented by a three-digit number. The code represents occupations rather than industrial sectors, e.g., an engineer working in a steel factory is included in sector 0 – professional/technical workers – and not in sector VII or VIII, which together include all blue-collar workers in the same factory. The first digit refers to one of 10 major occupational sectors (0–9), with the following figures representing increasingly detailed levels of disaggregation in accordance with the specific job. Hence, this categorisation enables manual to be distinguished from non-manual occupations, which often require longer education and are associated with higher socio-economic status. The two production sectors (VII+VIII) were pooled and then joined with Sector V (Mining/quarrying), which had a very low number of cases. Rurality was measured by size of town of residence in 1970 (<2000, 2000–20,000, 20,001–100,000 and >100,000 inhabitants). The exact number of person-years that each subject contributed to the study was allocated to the corresponding cells of the variables of stratification, namely: occupational sector; county and rurality; five-year age-group; and 5-year calendar period. Age and period were time-dependent variables.

Log-linear Poisson models were fitted to estimate relative risks (RRs), assuming that the observed number of cases was distributed in each stratum as a Poisson variable, with person-years as offset. In a first step, a Poisson model for all cases of cutaneous melanoma, adjusted for sex, age, period, occupational sector and town size, was used to group Swedish counties into three, geographically meaningful areas, according to whether their RRs were under 0.85 (Northern Sweden), from 0.85 to 1.15 (Central Sweden), or over 1.15 (Southern Sweden); thus, this new variable could be considered as a surrogate for regular environmental sun exposure. Additional log-linear Poisson models were then fitted to estimate adjusted relative risks for each gender and anatomical site separately, with respect to occupational sector and town size, adjusted for geographical area, age group and period of diagnosis.

## Results

Across follow-up, a total of 6187 cases of cutaneous melanoma were observed among men, with trunk accounting for 51%, head/neck and upper limbs for 12% each, and lower limbs for an additional 11%. Among women, 3598 cases of cutaneous melanoma were diagnosed, with lower limbs being the most frequent location (38%), followed by trunk (23%), upper limbs (18%) and finally, head/neck (11%).

Table 1 summarises the main results. Overall, the RRs for different sectors depict a socio-economic dichotomy in men, with excess risk in sectors 0–III (white-collar workers) and lower risks in production sectors, agriculture and transport. Sector IX (Services and military work), which includes an heterogeneous group of occupations, such as civilian protective service workers – policemen, fire-fighters, ...-, members of the armed forces and building caretakers, presents an intermediate position. Sectors 0–III also show risk excesses in trunk, and a negative association with production/mining and agriculture, forestry and fishing works. Upper and lower limbs had a similar pattern, with the exception of sector III (sales work).

**Table 1:** Relative Risk of cutaneous melanoma by occupational sector and rurality in a Swedish cohort.

		All cases			Head and neck			Trunk			Upper limbs			Lower limbs		
Occupational sector**	Py.	C	RR	95% CI	C	RR	95% CI	C	RR	95% CI	C	RR	95% CI	C	RR	95% CI

Men																
0 Professional/technical work.	6207331	1403	1.16	1.10 – 1.23	142	1.09	0.91 – 1.29	720	1.16	1.08 – 1.26	189	1.22	1.05 – 1.42	164	1.12	0.95 – 1.31
I Admin/managerial work.	1130346	333	1.27	1.15 – 1.41	31	1.02	0.74 – 1.40	166	1.23	1.07 – 1.42	47	1.41	1.09 – 1.84	43	1.44	1.09 – 1.89
II Bookkeeping & clerical work.	1490061	351	1.14	1.03 – 1.25	34	0.96	0.71 – 1.31	188	1.19	1.05 – 1.36	46	1.17	0.89 – 1.52	45	1.22	0.93 – 1.60
III Sales work.	2542799	576	1.10	1.02 – 1.19	67	1.14	0.91 – 1.43	311	1.16	1.04 – 1.29	72	1.07	0.86 – 1.34	64	1.03	0.82 – 1.29
IV Agric./forestry/fishing	3303350	504	0.77	0.70 – 0.85	98	0.99	0.79 – 1.24	217	0.67	0.59 – 0.77	64	0.79	0.61 – 1.02	48	0.73	0.55 – 0.98
VI Transport & communic.	2849849	488	0.88	0.81 – 0.95	54	0.85	0.66 – 1.09	275	0.97	0.86 – 1.08	57	0.80	0.63 – 1.02	52	0.79	0.62 – 1.02
V-VII-VIII Production/mining	14135070	2167	0.78	0.74 – 0.82	309	0.90	0.79 – 1.03	1107	0.79	0.74 – 0.84	255	0.71	0.62 – 0.82	231	0.71	0.61 – 0.82
IX Services & military work.	1700362	365	1.02	0.93 – 1.12	46	1.08	0.83 – 1.42	180	0.99	0.87 – 1.13	47	1.03	0.79 – 1.34	51	1.21	0.94 – 1.56
Women																
0 Professional/technical work.	4774089	858	1.10	1.02 – 1.19	68	0.86	0.66 – 1.11	221	1.27	1.06 – 1.52	156	1.12	0.94 – 1.34	328	1.11	0.97 – 1.26
I Admin/managerial work.	211219	39	1.03	0.78 – 1.36	4	0.92	0.39 – 2.20	5	0.63	0.29 – 1.36	14	2.02	1.26 – 3.23	14	0.98	0.62 – 1.56
II Bookkeeping & clerical work.	4333725	799	1.12	1.03 – 1.22	58	0.83	0.63 – 1.10	197	1.23	1.02 – 1.48	154	1.22	1.02 – 1.47	309	1.13	0.98 – 1.29
III Sales work.	2589201	467	1.00	0.91 – 1.10	50	0.90	0.67 – 1.20	108	1.11	0.90 – 1.37	84	0.97	0.78 – 1.20	181	1.03	0.88 – 1.20
IV Agric./forestry/fishing	968652	173	0.98	0.84 – 1.15	33	1.32	0.90 – 1.94	40	1.19	0.86 – 1.65	33	0.89	0.63 – 1.25	53	0.86	0.66 – 1.13
VI Transport & communic.	7404688	142	1.12	0.96 – 1.31	18	1.37	0.89 – 2.11	26	0.94	0.65 – 1.34	18	0.79	0.52 – 1.19	66	1.37	1.09 – 1.72
V-VII-VIII Production/mining	2345706	381	0.86	0.77 – 0.95	52	0.94	0.71 – 1.26	89	0.99	0.79 – 1.24	57	0.68	0.53 – 0.88	137	0.83	0.70 – 0.98
IX Services & military work.	4845690	739	0.84	0.77 – 0.91	116	0.99	0.79 – 1.24	146	0.83	0.68 – 1.01	132	0.79	0.65 – 0.95	265	0.82	0.71 – 0.94

Town size (pop.)																

Men																
Under 2,000 inhabitants*	9949869	1463	1.00		243	1.00		709	1.00		177	1.00		140	1.00	
2,000–20,000	8178533	1415	1.17	1.08 – 1.27	164	0.96	0.77 – 1.19	724	1.17	1.05 – 1.31	187	1.27	1.02 – 1.59	173	1.43	1.13 – 1.82
20,001–100,000	8382606	1698	1.37	1.26 – 1.48	199	1.14	0.93 – 1.41	869	1.38	1.24 – 1.53	214	1.41	1.13 – 1.76	201	1.61	1.27 – 2.04
Over 100,000	6848160	1611	1.31	1.21 – 1.42	175	1.02	0.82 – 1.28	862	1.34	1.20 – 1.50	199	1.31	1.04 – 1.65	184	1.53	1.20 – 1.96
Women																
Under 2,000 inhabitants*	4442561	707	1.00		97	1.00		160	1.00		138	1.00		242	1.00	
2,000–20,000	4995511	832	1.08	0.97 – 1.20	95	1.09	0.80 – 1.49	198	1.11	0.89 – 1.39	145	0.95	0.74 – 1.22	309	1.13	0.94 – 1.35
20,001–100,000	5850561	1010	1.14	1.03 – 1.27	109	1.07	0.79 – 1.45	235	1.16	0.93 – 1.44	168	0.95	0.75 – 1.22	381	1.24	1.04 – 1.47
Over 100,000	5520117	1049	1.07	0.96 – 1.19	98	0.80	0.58 – 1.10	239	1.13	0.90 – 1.42	197	0.98	0.77 – 1.26	421	1.23	1.03 – 1.46

RR: Relative risk Py: person-year CI: Confidence interval C: Cases

Models included both town size and occupational sector. All risks were also adjusted for age, period of diagnosis and geographical distribution

* Reference category

** Reference: average rate for all sectors

This socioeconomic dichotomy of risk was not as clear among women, in that only professionals (sector 0) and bookkeeping/clerical workers (sector II) displayed a higher risk of melanoma, with the lowest risks being observed for the production and services sectors. Most women within sector IX (Services) are kitchen maids, nursemaids, housekeeping service workers or cleaners, which can be considered low socioeconomic status jobs. Site analysis reflected this general pattern, but also showed a significant risk excess in administrative and managerial workers specifically limited to upper limbs melanomas, and in transport and communication works in lower limbs. Trunk did not present significant differences of risk in lower socioeconomic sectors.

However, the most noteworthy result in site analyses was that both sexes shared a certain uniformity of risk for head/neck melanoma, which did not vary by occupational sector.

Insofar as rural/urban distribution was concerned, significant differences were observed among men, with a small increase in risk in towns of 2,000–20,000 inhabitants, and higher RRs in larger towns, though this pattern mainly reflected the distribution observed in trunk melanoma. Among women, the rural/urban gradient was confirmed in legs only. Once again, the effect of town size seemed irrelevant for head/neck in both sexes.

## Discussion

One of the clearest results in our analysis is the specific risk pattern shared by both sexes for head/neck melanoma, in which the influence of socio-economic class and town-size is negligible. Interestingly, this is the only location with a similar proportion of cases in males and females. These results are coherent with regular unintentional sun exposure, and reinforce the previously suggested specificity of the epidemiological – and perhaps biological – profile of head/neck melanoma [23-27], with a higher percentage of lentigo malignant melanoma, an older age at diagnosis [25], and a higher proportion of cases with skin damage associated with chronic sun exposure [23].

In the other sites, our results confirm the well-known relationship between socio-economic class and melanoma risk in men [28], indicating that white-collar sectors registered a clear excess risk of melanoma in trunk, and upper and lower limbs. These results agree with those also reported for Sweden by Hemminki et al [29,30], which found that CM displayed one of the strongest, long-standing associations with higher educational levels across the sexes, an increased risk among professionals and a lower risk in agriculture, with differences being more intense in men versus women.

We also found a weaker association between melanoma and social class among women, where some heterogeneity also appeared. The risk excess found in transport and communication sector in lower limbs cannot probably be attributed to socioeconomic differences, and might be related with the higher incidence observed in specific occupations, such as motor-vehicle or tram drivers and telephone operators [11].

Occupation and residence in 1970 are assumed to be unchanged for the follow-up period of the subjects, as we have no individual information on changes vis-à-vis our cohort, something that could be regarded as a limitation of this study. However, this might be less significant in an analysis centred on occupational sectors rather than on risks posed by specific jobs. There are also ecological data showing that in Sweden the employment market was quite stable during the study period. According to Oyer et al [31], in 1974 over 92% of white-collar workers and over 88% of blue-collar workers in private firms had been employed for at least three years in the firm for which they were then working; job change became less common in Sweden from 1974 to 1982 and then became dramatically more common by 1990. The stability seems to have been more relevant in white-collar workers. Lazear et al [32] reported that in the 1971 Swedish workers cohort, about half of all white-collar workers remained in the same occupation over the 20-year period, suggesting a strong attachment to occupations for most of these, and that no gender-related differences were in evidence. In contrast, from 1974 to 1982 the number of blue-collar workers decreased because these workers moved over to the white-collar sector, part-time work or the public sector [31]. Average white-collar wages dropped by 8.5%, while blue-collar wages dropped by only 3.4%, thereby reducing the gap between blue- and white-collar wage levels. The above-mentioned trend mainly reduced socio-economic differences among the groups during our follow-up period, yet even with this increased homogenisation there was an association between melanoma and occupational sector.

The significant correlation reported in Sweden between high travelling frequency to sunny countries and higher social classes [2] or high education [33] might explain the gradation observed, as the increased risk present in normally covered anatomical locations among white-collar workers, particularly in office jobs [2,34], has been attributed to intermittent leisure-time sun exposure [35]. However, the lack of direct personal data on sun-exposure patterns, which constitutes the main limitation in this analysis, prevents us from identifying whether any other factor associated with social class, apart from UV exposure, might also account for the association observed.

The different distribution of risk by gender could reflect a different labour structure: over 50% of women versus 1/3 of men belong to sectors 0–III, and low-income female jobs are in all likelihood included under sales/clerical work, whereas the worst-paid men work in production sectors. Thus, job category might be less accurate for estimating socio-economic level in women than it is in men. Indeed, other authors [1] have preferred husbands' occupations for measuring female social class. However, this approach is losing validity [36], given the increased participation of women in the workforce – close on 50% in Sweden in the 1970s [37] – as well as the rise in female-headed households.

An alternative explanation of these results could be a greater uniformity of risk among females. Tanning habits such as outdoor sunbathing [38] or sun-bed use [39] are widespread among the entire female Swedish population, much more so than they are among men. It should be also noted that our cohort solely included employed persons. Whilst this restriction enabled us to use individual occupation as a surrogate measure for socio-economic class for both sexes, it also implied that the cohort had a higher proportion of men, since only half of the female population in the selected age-groups was occupied, in contrast with Swedish men, who were active in almost all cases [37]. Within their respective populations, working females, who differ from home workers in many lifestyle-related factors [40], could constitute a more homogeneous subgroup than do working men.

The different melanoma risk patterns by town size for males and females, something that cannot be attributed to occupational misclassification, might also support this possibility. Although the association between melanoma and rurality has not been studied in great detail, lower risks are generally found in small towns [41], which may probably reflect behavioural differences [33]. Insofar as town size is concerned, in Sweden the propensity to migrate decreased slightly during our follow-up period [42]: in all, from 1971 to 1996 the number of people migrating across municipal borders decreased from 5.1% to 4.5% of the population. Migration led to dispersion of the population in the 1970s. Thereafter, the larger urban areas had a net inward-migration in the first half of the 1980s but this was followed by a net outward-migration in the latter half of the decade.

Again, town size in Sweden has been shown to be related to frequency of foreign travel, estimated on the basis of passport use [8]. Aase et al [43] also described higher incidences in Norwegian towns of over 10,000 inhabitants, in spite of a certain tendency towards convergence over time. A similar result was likewise found among both sexes in The Netherlands [44]. It should be noted that other authors have not observed a clear relationship between melanoma and town size [45].

Nowadays, low-cost airlines and all-inclusive package holidays have made trips to sunny destinations affordable for many persons. Sunbed use has also become more common. This increasingly generalised UV exposure may perhaps lead to a more uniform distribution of risk by social class or town size in the future. Moreover, the association with social class may even be reversed, should sun-protective behaviour become the norm among higher social strata, much in the same way as has happened with tobacco use.

## Conclusion

The size of our cohort and the large number of cases registered rendered it possible for us to study and compare melanoma incidence patterns by socio-economic class and rurality between the sexes and among sites, simultaneously. While differences in risk between men and women could suggest greater homogeneity in UV-exposure behaviour among women, the uniform risk pattern in head and neck melanoma present in both sexes might support the coexistence of different aetiological pathways, related to anatomical site. Our results serve as a reminder that site and gender should always be considered in cutaneous melanoma research.

## Competing interests

The author(s) declare that they have no competing interests.

## Authors' contributions

BP, PG & MP conceived the study, GL, VL, NA & BP performed the statistical analyses and BP drafted the manuscript. All authors have read and approved the final manuscript.

## Pre-publication history

The pre-publication history for this paper can be accessed here:


